# Determining Predictors of Weight Loss in a Behavioral Intervention: A Case Study in the Use of Lasso Regression

**DOI:** 10.3389/fpsyt.2021.707707

**Published:** 2022-02-03

**Authors:** Carly Lupton-Smith, Elizabeth A. Stuart, Emma E. McGinty, Arlene T. Dalcin, Gerald J. Jerome, Nae-Yuh Wang, Gail L. Daumit

**Affiliations:** ^1^Johns Hopkins Bloomberg School of Public Health, Baltimore, MD, United States; ^2^Johns Hopkins School of Medicine, Baltimore, MD, United States; ^3^Department of Kinesiology, Towson University, Towson, MD, United States

**Keywords:** prediction models, behavioral interventions, Lasso regression, obesity, serious mental illness (SMI), bipolar disorder, major depressive disorder

## Abstract

**Objective:**

This study investigates predictors of weight loss among individuals with serious mental illness participating in an 18-month behavioral weight loss intervention, using Lasso regression to select the most powerful predictors.

**Methods:**

Data were analyzed from the intervention group of the ACHIEVE trial, an 18-month behavioral weight loss intervention in adults with serious mental illness. Lasso regression was employed to identify predictors of at least five-pound weight loss across the intervention time span. Once predictors were identified, classification trees were created to show examples of how to classify participants into having likely outcomes based on characteristics at baseline and during the intervention.

**Results:**

The analyzed sample contained 137 participants. Seventy-one (51.8%) individuals had a net weight loss of at least five pounds from baseline to 18 months. The Lasso regression selected weight loss from baseline to 6 months as a primary predictor of at least five pound 18-month weight loss, with a standardized coefficient of 0.51 (95% CI: −0.37, 1.40). Three other variables were also selected in the regression but added minimal predictive ability.

**Conclusions:**

The analyses in this paper demonstrate the importance of tracking weight loss incrementally during an intervention as an indicator for overall weight loss, as well as the challenges in predicting long-term weight loss with other variables commonly available in clinical trials. The methods used in this paper also exemplify how to effectively analyze a clinical trial dataset containing many variables and identify factors related to desired outcomes.

## Introduction

The prevalence of obesity among individuals with serious mental illness (SMI) is substantially higher than that in the overall population (1–6). This heightened burden of obesity contributes to various health risks, such as hypertension and diabetes mellitus (7–9). People with SMI have higher levels of physical inactivity and poorer diets than the general population (10–12). Combined with psychotropic medications that can cause weight gain as a side effect and possible impairments of memory and functioning, physical inactivity and higher calorie, lower nutrient dense diets in the SMI population can be difficult to combat in order to lose weight (13). The heightened health risks and obesity experienced by the SMI population combined with their potential functional limitations indicate the necessity for behavioral weight loss interventions tailored specifically to this group. With a tailored intervention, it is worth investigating which factors will predict successful weight loss in this population. Identifying early predictors of long-term weight loss can help in the planning of future interventions including potential mid-course adjustments to ensure additional support is provided to those who might need the assistance.

Although fewer behavioral weight loss interventions have been implemented for the SMI population compared to the rest of the population, some programs have been carried out with successful results (14–16). A systematic review of 80 studies involving interventions for individuals with SMI that addressed overweight or obesity found high strength of evidence for behavioral interventions (14). Including the ACHIEVE trial, there were 22 randomized controlled trials and 15 observational studies that investigated the effects of behavioral weight loss interventions on weight, and the overall evidence showed that the interventions resulted in improved weight loss.

The ACHIEVE trial was conducted from 2009 to 2011 with the partnership of 10 community psychiatric rehabilitation programs and 291 participants randomized to the intervention or control group (16). The behavioral weight loss intervention employed in this study was tailored to the SMI population and involved group and individual weight-management sessions and group exercise sessions. The overall intervention lasted 18 months, with 6 months of more intensive intervention. The study ultimately found a significant increase in weight loss over the study period for the intervention group, and this group had significantly more weight loss compared to the control group. The average weight loss at 18 months (7 lbs) was similar to that found in other weight loss trials in the general population (1).

The goal of the present paper is to identify predictors of 18-month weight loss in the intervention group. Ultimately, if key predictors are discovered, these variables can aid in the identification of individuals who are likely to experience weight loss with the current program and those who need greater assistance in losing weight. For example, in the implementation of future interventions, individuals who do not have initial weight loss success could potentially receive adaptations of the intervention based on their characteristics at a given time point, a design strategy used in adaptive clinical trial designs (17).

The methods are illustrated using this weight loss example; however, they can apply much more broadly. The paper thus also serves a second purpose, which is to illustrate the use of Lasso regression to identify the strongest outcome predictors among a large set of potential background characteristics (18). Such insights can help identify the characteristics of individuals most likely to experience specific outcomes, which can help with tailoring interventions or identifying those individuals most at risk for poor outcomes. Like standard linear/logistic regression, Lasso regression models can be used to model an outcome of interest as a function of predictors, and the Lasso model generates regression coefficients, with their usual interpretation. A key benefit, however, is that Lasso regression can handle a much larger set of potential predictors than can traditional regression approaches. Lasso regression has been implemented in many statistical publications and a significant number of biomedical studies [e.g., (19–22)], but has been utilized less often in behavioral research (23). Specifically, many behavioral science publications use conventional variable selection procedures, such as stepwise selection, which can lead to model overfitting (23). Lasso regression can select from a large set of variables those that are the most impactful, as well as not overly related to one another. Lasso is therefore a useful tool for settings with a large set of potential predictors to identify those most predictive of outcomes.

## Methods

### Achieve Trial Design

The ACHIEVE trial included overweight or obese adults who attended 10 community psychiatric rehabilitation day programs in central Maryland (1, 16). Because the original ACHIEVE results showed that intervention participants did lose weight compared to controls, the primary methodology of the present study focuses on the statistical analysis of the predictors of this weight loss. The subset of data analyzed included participants assigned to the weight loss intervention (*n* = 144), as the present research is most interested in what predicted weight loss in individuals who received the behavioral intervention. Before analyses were conducted, individuals with missing outcomes were removed from the dataset (4.86%), leaving 137 individuals for analysis.

### Exploratory Analysis

Exploratory data analysis was first performed to investigate the relationships between key participant variables and weight loss. The first step in the analysis was determining which variables to include as potential predictors of weight loss, as the ACHIEVE trial gathered information on over 1,000 features. Available features included demographic information, diagnosis and medication, medical history, physical and laboratory measures, and questionnaires measuring health behaviors, weight loss history, eating and physical activity habits, psychiatric symptoms, social support, self-efficacy, and more, in addition to data collected during the intervention such as weigh-ins and session attendance (24, 25). A comprehensive table of data collection measures and trial registration information can be found in the trial protocol paper (16). Variables were selected primarily based on expert opinion and literature review (4–6), with some preliminary investigations into the data using correlations, *t* tests, and scatterplots to highlight potential relationships between variables and weight loss. From expert opinion, the following categories of variables were elected to be included: baseline demographics, diagnosis and medication, physical health, weight efficacy and self-efficacy, intervention attendance, weight loss history, and social support. For many of these categories, questionnaire subscales were utilized, as they consisted of summed or averaged scores from responses to multiple individual questions. The final list of variables included as potential predictors can be found in the Appendix. Predictors were used from baseline and from 6 months if available. Additionally, when both baseline and 6-month data were available for the same variable, the difference between the two quantities was also included as a potential predictor. Variables were standardized for easier coefficient interpretation to have mean 0 and standard deviation 1, with the exception of categorical variables (i.e., psychiatric diagnosis). The outcome of interest in this analysis was a binary indicator of whether an individual lost at least five pounds of weight (1) or did not (0) across the 18 months of the intervention. This outcome was selected because the original ACHIEVE trial was powered to find 4.5 pounds of weight loss. Furthermore, 5 pounds were selected as an outcome based off of Stevens and colleagues' recommendation of weight maintenance being defined as less than three percent change of body weight (26). Based on the average baseline body weight of participants exposed to the intervention in the ACHIEVE study, three percent of this body weight is close to 5 pounds, so this cutoff of weight loss was considered to be enough to be defined as notable weight loss.

### Missing Data

The next step after exploratory data analyses is evaluating and handling missing data. Based on Graham's recommendation, variables with less than five percent of their data missing were treated with mean imputation, in which the missing value was replaced with the average value of the non-missing data for that column (27). For variables with more than five percent of their data missing, multiple imputation was performed using the mice package in R (28). This procedure fills the missing data with values based on a model created using other known values of other variables, with a random aspect. Twenty imputations were performed, creating twenty datasets. After imputation, 6-month vs. baseline difference variables were incorporated into the dataset as other possible predictors. Example code for the imputation procedure, as well as the Lasso regression and classification tree to follow can be found in the Appendix.

### Lasso Regression

With missing data imputed, Lasso regression could be effectively performed. Lasso regression is similar to multiple linear/logistic regression but uses regularization, in which the loss function to minimize now includes an added regularization parameter multiplied with a penalty function (23). This penalty term reduces overfitting and the potential for very large coefficients. In Lasso regression, the penalty constrains the sum of the absolute value of the coefficients so that they are less than some constant. This upper bound on the sum of the coefficients causes coefficients of some variables to be exactly 0. So, the approach determines a sparse model that highlights the variables that have a strong relationship with the outcome of interest, and selects coefficients that should be set to 0, thus preserving degrees of freedom of the model overall. The regularization parameter involved in the penalty term can be determined using k-fold cross-validation, in which the data is randomly split into k groups. Then, k iterations are performed in which each group is sequentially left out of the model fitting and used as testing data after the model is fit on the remaining data. Each model created in the iterative process is evaluated to determine the best value of the regularization parameter.

Lasso regression was performed using a set of 68 preselected variables as predictors and the binary 18 month weight loss indicator (lost at least 5 pounds or did not) as the outcome. The MAMI package in R was used, which allows for the set of imputed datasets to be input, along with a model specifying the outcome and a possible set of predictors (29). Five-fold cross-validation was performed during the model fitting to determine the best value of the regularization parameter. The regression procedure then output mean coefficients with confidence intervals for each variable based on the results from Lasso regressions performed on the 20 imputed datasets. Because of this process of averaging coefficients from each imputed dataset, the average coefficients were affected by how many imputed datasets selected the variable in the Lasso regression. A detailed example of how to implement Lasso regression in behavioral science research is outlined by McNeish in his paper (23).

In order to visualize the predictive models, classification trees were formed for the imputed datasets using the rpart package in R (30). Classification trees split the data based off of certain values of key variables to visualize where individuals differ in their successful weight loss or lack thereof. These differences can be identified to use as decision points in an adaptive design; if an individual has a score above or beneath a certain cutoff of a key variable, an adaptation of the intervention might be necessary. It is important to note that classification trees are specific to the dataset and might overfit (31). Furthermore, methods for classification and regression trees do not exist for multiply imputed data, so a random handful of the imputed datasets were used to form classification trees. The classification trees formed were relatively consistent across datasets, meaning that most trees displayed the same branching rules; one example tree is shown in the following section.

Finally, given the preliminary results found from the Lasso and classification trees, a *post-hoc* analysis was performed investigating predictors of 6-month weight loss. This analysis no longer used Lasso but was a multiple logistic regression, since the purpose of this model was to look at various preset predictors rather than identify the most influential ones. Furthermore, only mean imputation was used, as no variables had more than five percent missingness.

## Results

### Descriptive Statistics

Of the original 291 participants in the ACHIEVE study, 144 were assigned the intervention. Of these 144 participants, 137 had a weight outcome at 18 months and were used in the analysis. Seventy-one (51.8%) individuals had an overall net weight loss of at least five pounds (2.3 kilograms) from baseline to 18 months (Table 1). In this group that lost at least five pounds across the entire study period, the mean age was 46.5 years, 49.3% of the group was male, and 59.2% were white and 32.4% black.

**Table 1:** Participant characteristics by weight loss category.

	**Lost at least 5 lbs after** **6 months**	**Did not lose at least 5 lbs after** **6 months**	**Lost at least 5 lbs after** **18 months**	**Did not lose at least 5 lbs after** **18 months**
Total**–**n	**53**	**84**	**71**	**66**
Age–mean ± sd	47.4 ± 12.1	46.5 ± 10.6	46.5 ± 11.9	47.2 ± 10.4
Male–%	56.6	46.4	49.3	51.5
Baseline weight–mean ± sd	223.3 ± 45.4	221.6 ± 49.6	221.1 ± 47.6	223.6 ± 48.5
Race				
White–%	56.6	56.0	59.2	53.0
Black–%	35.8	36.9	32.4	40.9
Other–%	7.5	7.1	8.5	6.1
Hispanic–%	5.7	2.4	5.6	1.5
Primary psychiatric diagnosis				
Schizophrenia–%	32.1	31.0	26.8	36.4
Schizoaffective–%	28.3	29.8	35.2	22.7
Bipolar disorder- %	15.1	21.4	19.7	18.2
Major depression–%	18.9	9.5	15.5	10.6
Other–%	5.7	8.3	2.8	12.1
Number of medications–mean ± sd	7.3 ± 3.8	7.5 ± 4.3	7.2 ± 3.8	7.7 ± 4.4
Any antipsychotics–%	75.5	71.4	69.0	77.3
Baseline weight efficacy score–mean ± sd	110.5 ± 41.6	110.3 ± 35.7	109.4 ± 40.3	111.5 ± 35.5
Baseline self efficacy score–mean ± sd	29.0 ± 6.1	30.3 ± 6.1	29.0 ± 6.6	30.6 ± 5.4
Consider self to be overweight at baseline–%	83.0	82.1	80.3	84.8
Trying to lose weight at baseline–%	79.2	75.0	78.9	74.2
Trying to lose weight at 6 months–%	86.8	78.6	88.7	74.2
Importance of controlling weight at baseline (1–10)–mean ± sd	8.7 ± 2.2	8.9 ± 1.6	8.8 ± 2.0	8.8 ± 1.7
Confidence in ability to change weight (1–10) at baseline–mean ± sd	7.1 ± 2.9	7.5 ± 2.6	7.5 ± 2.8	7.2 ± 2.7
MOS social support score at baseline–mean ± sd	62.8 ± 22.8	64.2 ± 20.8	62.8 ± 21.3	64.6 ± 21.9

### Lasso Regression

To prepare the data for Lasso regression, missing data was first addressed. The majority of the variables had no missingness (*n* = 54, 79.4%). Seven variables (10.3%) had missingness of <5%; six of these were handled using mean imputation and one using mode imputation because it was categorical. The remaining seven variables (10.3%) were multiply imputed because they had missingness ranging from 7.3 to 8.8%. More information about missingness and imputation can be found in the Appendix 6.2. Table 2 shows the results of the Lasso regression performed with 68 predictors and the outcome of at least five pounds of weight loss (1) or less than five pounds of weight loss (0) at 18 months. The table displays the mean coefficient estimates and their confidence intervals based on the multiply imputed datasets. The variables that the Lasso selected for this model were weight loss from baseline to 6 months, whether the participant was trying to lose weight or not at 6 months, whether the participant could rely on social support a lot or not at 6 months, and whether the primary psychiatric diagnosis was in the category of “Other”. From the model coefficients, fitted probabilities of losing at least five pounds of weight loss at 18 months were calculated, and predicted weight loss statuses were assigned based on whether each predicted probability was >0.5 (assigned a status of “lost at least five pounds at 18 months”) or ≤ 0.5 (assigned a status of “did not lose at least five pounds at 18 months”). In comparing the fitted classifications according to the model with the true classifications of weight loss, the model had 75.2% accuracy.

**Table 2:** Resulting model from Lasso regression.

	**Coefficient** **estimate**	**Std error**	**Lower CI**	**Upper CI**
Intercept	0.12	0.43	−0.72	0.95
Weight loss from baseline to 6 months	0.51	0.45	−0.37	1.40
Trying to lose weight at 6 months	0.01	0.26	−0.49	0.51
Can rely on social support a lot at 6 months	−0.05	0.46	−0.95	0.86
Primary psychiatric diagnosis of “Other”^*^	−0.14	0.90	−1.90	1.63

* *Primary psychiatric diagnosis categories were: Schizophrenia, Schizoaffective disorder, Bipolar disorder, Major Depressive Disorder, Other*.

Based on the magnitude of the coefficients, the weight loss from baseline to 6 months has the largest impact on 18 month weight loss out of the predictors selected in this model, and out of all of the predictors put into the Lasso. Specifically, the standardized coefficient of 0.51 for this variable indicates that, holding other variables fixed, 10.2 more pounds of weight loss at 6 months is associated with 67% higher odds of losing at least five pounds at 18 months. As previously described, these coefficients are based on the outcomes of Lasso regression performed on each imputed dataset. Therefore, some variables can have mean coefficients that are very close to zero, meaning that they were not selected for every dataset but were selected for at least one (29, 32). Furthermore, it is important to note that the coefficients have wide confidence intervals, implying that making strong conclusions about these variables might be difficult. All variables not shown in Table 2 were not selected for the Lasso regressions using any of the imputed datasets. These results indicate that 6-month weight loss is a dominant predictor of 18-month weight loss, while the other variables have limited predictive ability.

The same model was run with an outcome of at least 5% weight loss across 18 months (1) or <5% weight loss (0), and the results were very similar to those presented in Table 2. However, the only variable selected for this 5% weight loss model was weight loss from baseline to 6 months. Similarly, a model with an outcome of any weight loss across 18 months (1) vs. weight gain (0) was also run, and again, weight loss at 6 months was the only variable with a notable coefficient selected, and trying to lose weight at 6 months also came up.

### Six Month Weight Loss

**Figure 1:**
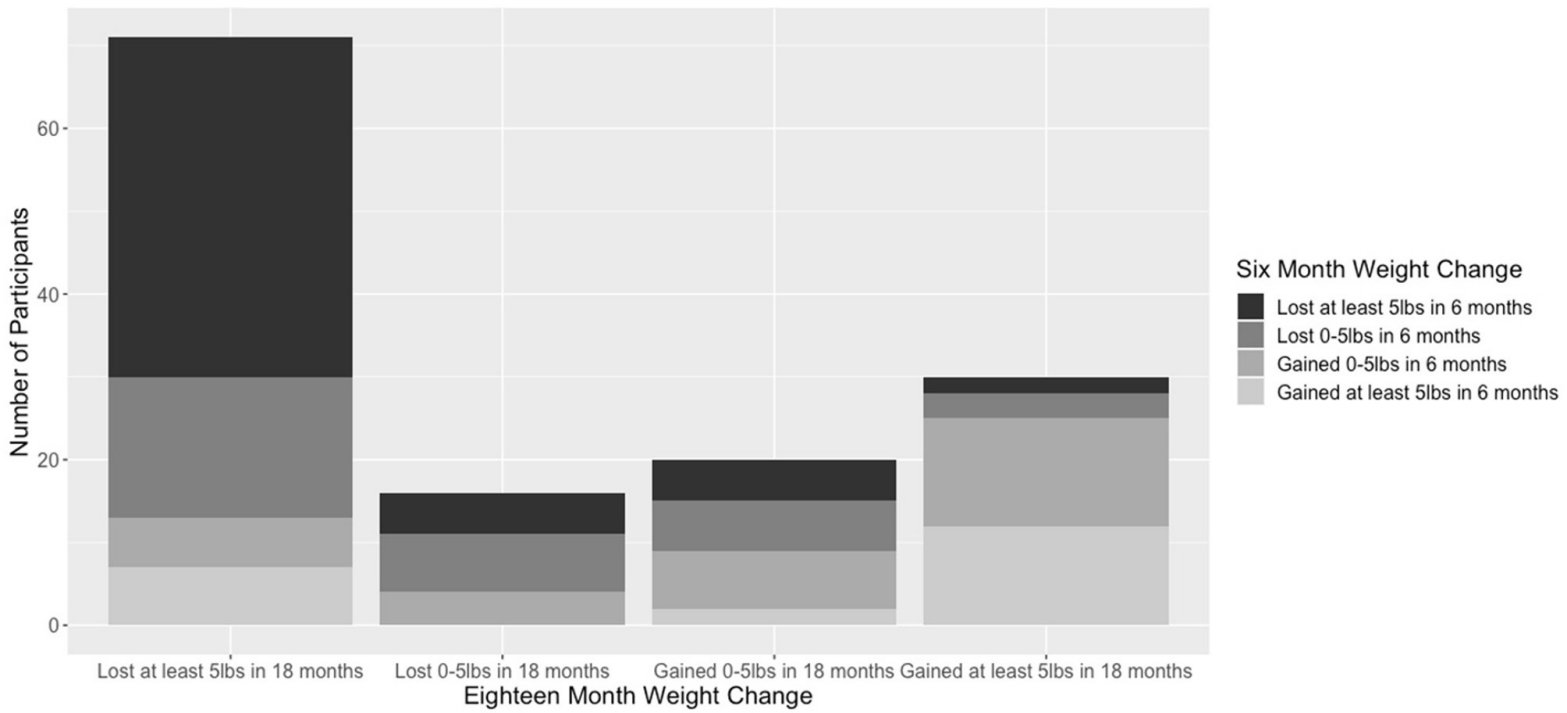
Participants in each category of 6-month weight change and 18-month weight change.

To further investigate the relationship between 6 month and 18 month weight loss, Figure 1 shows the number of participants in each category of 6 month weight change and 18 month weight change. The majority of participants lost at least five pounds in 18 months (*n* = 71), and of this group, 57.7% (*n* = 41) participants lost at least five pounds at 6 months. The correlation between weight loss at 6 months and weight loss at 18 months is 0.46 (*p* < 0.001).

Since the weight lost from baseline to 6 months was a key variable identified by the Lasso procedure, it is worth investigating which baseline factors predict this earlier weight loss. A regression without any multiple imputation was performed with the baseline variables used in the previous analyses, but this time looking at the outcome of a 6 month binary weight loss (1) or gain (0) indicator. The resulting coefficients from a multiple logistic regression are presented in the appendix (Appendix 6.4). This regression found that taking any antipsychotics (coefficient of −0.09) and identifying racially as Black or African American (coefficient of −0.19) both had *p* < 0.10, so these two variables had the strongest effects on six-month weight loss. Both variables indicate a lower odds of losing weight if present, meaning a Black individual and an individual taking antipsychotics were less likely to lose weight at 6 months.

### Classification Tree

**Figure 2:**
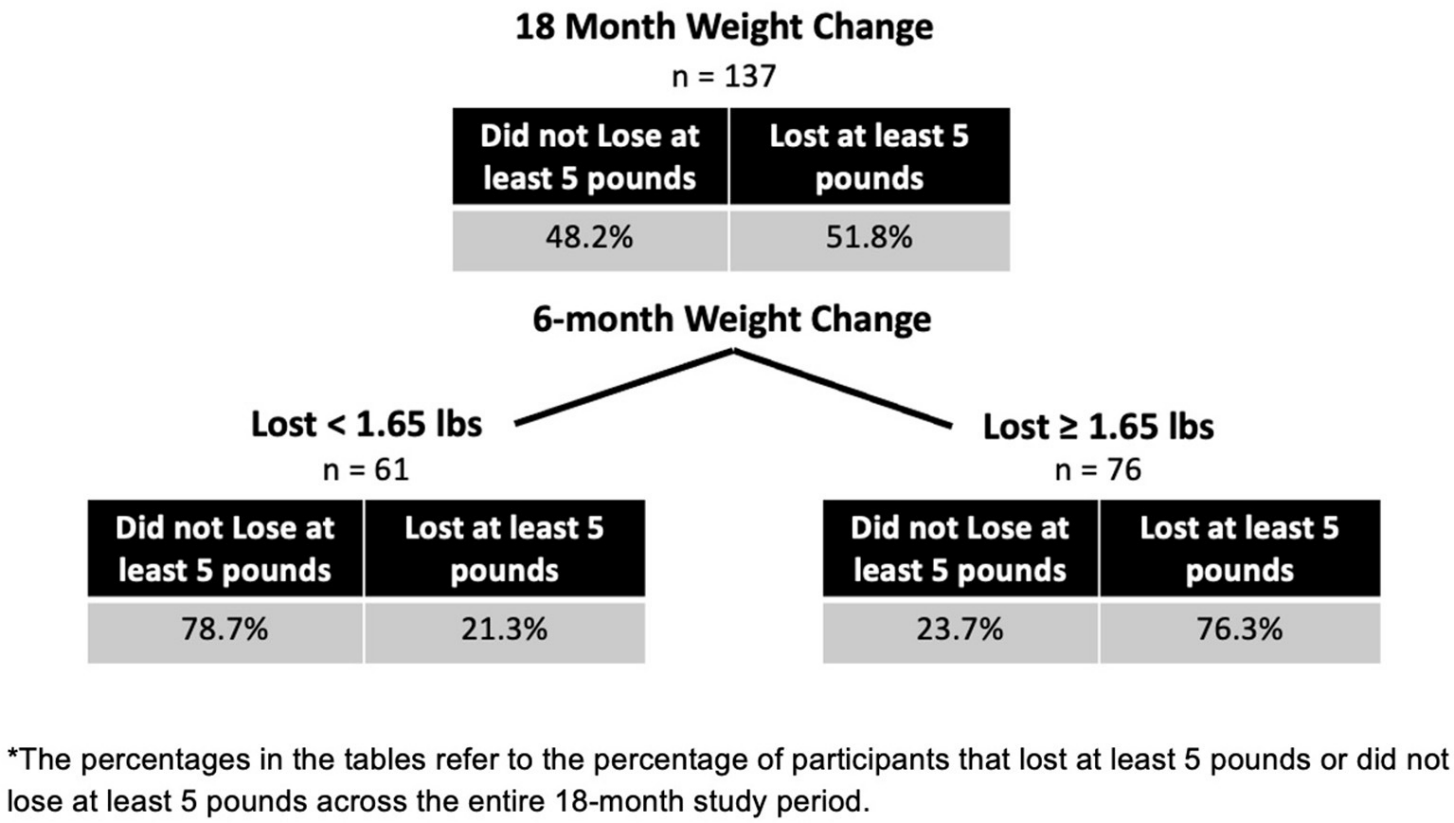
Classification tree for 18-month weight loss*.

After having selected the variables in a model of weight loss over 18 months, a classification tree was created that splits participants into whether they are likely to lose or not lose weight based on their values of key variables. Figure 2 is an example of a classification tree formed using one of the imputed datasets. The tree shows an example of how variables can be broken up into key decision points that show the likely outcomes of participants.

## Discussion

The goals of this paper were (1) to examine the possible predictors of weight loss in the ACHIEVE behavioral weight loss trial intervention group and (2) to illustrate the usage of Lasso regression as a tool for identifying outcome predictors in behavioral research. Although underutilized in behavioral research, the methods employed in this paper of Lasso regression and classification trees are effective tools for investigating key factors that predict an outcome, especially when there are many possible predictors. The results from these analyses help highlight which participants were “on the right track” in the intervention and which participants were not.

The ACHIEVE dataset included thorough assessments of participants' physical and mental health, as well as their behaviors, social support, and more. Similar to other behavioral interventions, the data collection led to a large number of variables. Traditional regression approaches are suboptimal when dealing with such large numbers of potential predictors. The shrinkage methods in Lasso allows for key variables to be identified, while limiting collinearities between key variables. Therefore, this approach can be very helpful in finding predictors of a given outcome in behavioral interventions and research. Although other methods such as Elastic Net and Ridge regression could be used to address these questions, Lasso was determined to be best suited to the study goals of finding specific variables that could be used to identify individuals who may benefit from further intervention and remove highly related variables.

It is also important to reflect upon the variables that were selected by the Lasso regression. Interestingly, with 68 potential predictors, only four were identified as predictors, and one had a strong coefficient. This result indicates a couple of possible interpretations. One view could be that it is very hard to predict weight loss in the SMI population; the potential predictors included a wide set of variables thought to be likely to predict weight loss, and very few were identified as predictors. Factors such as sample size could have impacted this result as well. Another view could be that the intervention used in the ACHIEVE study was broadly applicable to the entire SMI population. Demographic factors such as gender, race, and age were not identified as predictors of weight loss. One interpretation of this is that the ACHIEVE intervention was widely applicable for a range of participants with SMI, without strong predictors of which participants most benefit. This view is supported in work done by Alexander and colleagues who found support that the ACHIEVE trial is applicable across broad populations of individuals with SMI (33). The missingness in the data likely did not affect the results too drastically because mean imputation was performed on six variables that did not have major outliers, and multiple imputation was performed on seven variables (Appendix 6.2).

This analysis showed that early weight loss is a strong predictor of later weight loss, so early monitoring of weight is important to identify individuals who will likely lose weight or not by the end of the intervention. Of the 53 individuals in this sample who lost at least five pounds at 6 months, 77% of them lost at least five pounds at 18 months, while the remaining 23% did not and therefore gained at least some weight back. Therefore, although some individuals do not lose weight by the end of the intervention even if they lose some weight at the beginning, weight loss at 6 months is overall a helpful indication of an individual's eventual weight loss at 18 months. This result is consistent with findings from weight loss literature in the general population; participants who lost weight quickly or lost weight in the first month or couple of months of behavioral treatment had higher likelihoods of losing a significant amount of weight by the end of the intervention months or even years later (34, 35).

The exclusion of the remaining 64 variables in the results of Lasso could have been due to variability in their relationships with 18 month weight loss, collinearities with the variables that were identified as predictors, or other reasons. Variables such as session attendance, weight loss self-efficacy, and social support were not identified as predictors, which was a surprising result. Future work could pursue these variables in a different sample, or examine some in the control group of the ACHIEVE study itself to see if they might be predictors of weight loss in the group not exposed to the intervention. This analysis shows the challenge in identifying predictors of weight loss, but as previously mentioned, this could be indication of an intervention that is broadly applicable and works similarly for everyone involved.

The classification tree in Figure 2 gives an example of how researchers can use trees to develop insight into possible decision rules for an adaptive trial design, like a SMART design (17). From this tree, an intervention could focus on 6-month weight loss and expose the group that lost <1.65 pounds (0.75 kilograms) to a stronger adaptation of the intervention. In a SMART design, participants are randomly assigned to certain adapted treatments at set time points during an intervention, based on their initial weight loss or answers to some key questions. With the combination of Lasso regression and a classification or regression tree, the two analyses work together to show which variables might be important indicators of a need for an adaptive intervention, and at what level of the key variables should a person be given more in-depth focus.

Furthermore, when developing an adaptive intervention, variables that can be intervened upon and adjusted should be identified. In this paper, for example, an adaptive intervention could target weight loss after 6 months to try and change that trajectory, or it could focus on improving the individual's motivation to lose weight at 6 months. On the other hand, some variables are unchangeable; in the 6-month analysis, being Black had one of the strongest effects on weight loss, which is not malleable by an intervention. Therefore, determining not only which variables have significant relationships with the outcome, but also which variables can actually be intervened upon is vital to developing an adaptive intervention.

This study exemplifies how to employ a relatively uncommon methodology in behavioral research to identify predictors of weight loss. Strengths included that missing data was rare, and the data was collected in a very comprehensive and thorough way. There were some limitations to the analysis that could be improved upon in future work. First, as mentioned before, the methods used in this paper help identify who had lost weight by month six and who might have benefited from an adaptive intervention at that time, but the methods are not easily able to explore what intervention is needed for these individuals. More work could be done to identify adaptive interventions for this population, such as whether individuals should receive assistance with physical activity, mental health, health-related attitudes or behaviors, or other possible focal points.

With the large number of thorough and well-collected measures present in the ACHIEVE study, the analyses performed in this paper demonstrated both the challenges in predicting weight loss, and the importance of weight loss throughout an intervention as a predictor of weight loss at the end. The methods used in this paper are broadly applicable as a strategy to effectively deal with a large dataset and can help highlight important factors related to the study outcome.

## Data Availability Statement

The datasets presented in this article are not readily available because use could identify study participants. Requests to access the datasets should be directed to Gail Daumit, gdaumit@jhmi.edu.

## Ethics Statement

The studies involving human participants were reviewed and approved by Johns Hopkins Medicine Institutional Review Board. The patients/participants provided their written informed consent to participate in this study.

## Author Contributions

Analysis plan and study design worked on by ES, GD, EM, N-YW, and CL-S. Data analyses carried out and paper draft written by CL-S and ES. Adjustments and follow-up recommended by AD and GJ. Final paper reviewed and edited by all authors.

## Funding

This work was supported by the National Institute of Mental Health [NIMH P50115842].

## Conflict of Interest

The authors declare that the research was conducted in the absence of any commercial or financial relationships that could be construed as a potential conflict of interest.

## Publisher's Note

All claims expressed in this article are solely those of the authors and do not necessarily represent those of their affiliated organizations, or those of the publisher, the editors and the reviewers. Any product that may be evaluated in this article, or claim that may be made by its manufacturer, is not guaranteed or endorsed by the publisher.

## Abbreviations

SMI: Serious Mental Illness.
