# Supplementary material for: Determining Predictors of Weight Loss in a Behavioral Intervention: A Case Study in the Use of Lasso Regression
Source: Front Psychiatry. 2022 Feb 3;12:707707. doi: 10.3389/fpsyt.2021.707707 (PMC8850776; doi:10.3389/fpsyt.2021.707707)
Supplement: Supplementary file 1 [file Data_Sheet_1.docx]

**6. Appendix**

*6.1 Predictors Included in Lasso Regression*

- Baseline demographics
  - Race
  - Gender
  - Hispanic or not
  - Age
  - Site
- Diagnosis and medication
  - Primary diagnosis (1=schizophrenia, 2=schizoaffective, 3=bipolar, 4=major depression, 5=other)
  - Number of psychotropic medications
  - Any antipsychotics
  - Any atypical antipsychotics
  - Benzodiazepines
  - Clozapine
  - Mood stabilizers
  - Antidepressants
- Physical information
  - Height
  - Baseline weight
  - Difference between 6 month and baseline weight
- Weight efficacy scores (at baseline, 6 months, and 6 month/baseline difference)
  - Total weight efficacy score
  - Negative emotions score
  - Availability score
  - Social pressure score
  - Physical discomfort score
  - Positive activities score
- Intervention attendance information at 6 months
  - Total number of days in extended absence after 6 months
  - Number of extended absence occurrences after 6 months
  - Sum of all three attended sessions for 6 months
  - Group weight management attendance percentage at 6 months
  - Group Exercise attendance percentage at 6 months
  - Individual weight in attendance percentage at 6 months
  - Weighted average attendance percentage at 6 months
- Weight loss history (at baseline)
  - Are you overweight for your height and age?
  - Have you tried to lose weight in the past?
    - If so, has it been through physical exercise, such as walking, swimming, or calisthenics?
    - Has it been through a comprehensive weight loss program with dietary changes, physical activity, and behavioral counseling?
    - Has it been through keeping a log or journal for eating or exercise?
    - Has it been through taking a prescription medication to lose weight?
  - Have you been overweight since childhood (before age 18)?
  - Have you gained weight gradually over the years?
  - Have you gained most of your excess weight in a short period of time?
  - Have you gained and lost weight many times over the years ("yo-yo")?
  - How much can you rely on family or friends for support and encouragement?  (0=not at all, 1=somewhat, 2=a lot) (also at 6 months)
  - On a scale from 0-10, how important is controlling your weight to you personally? (also at 6 months)
  - On a scale from 0-10, how confident are you that you can successfully change your eating and physical activity to control your weight? (also at 6 months)
  - Do any of the following have anything to do with your being overweight?
    - Eating because of emotions and stress
    - Family or relationship problems
    - Loneliness or loss of loved one
    - Difficulty with self control
  - Are you trying to lose weight now? (also at 6 months)
- Self-efficacy (at baseline, 6 months, and 6 month/baseline difference)
- MOS Social Support score at baseline (34-35)
  - Total MOS score
  - Emotional/information support score
  - Tangible support score
  - Affectionate support score
  - Positive social interaction score
- Social support and eating score at baseline
  - Encouragement score
  - Disencouragement score
- Social support and exercise score at baseline
  - Family participation score
  - Friends participation score
  - Family reward and punishment score

*6.2 Missingness*

| Variable with missing values | Percent missing | Method for handling missingness | Mean (for mean imputed data) | Median (for mean imputed data) |
| --- | --- | --- | --- | --- |
| On a scale from 0-10, how important is controlling your weight to you personally? (6 months) | 4.4% | Mean imputation | 8.6 | 10.0 |
| On a scale from 0-10, how confident are you that you can successfully change your eating andphysical activity to control your weight? (6 months) | 4.4% | Mean imputation | 7.3 | 8.0 |
| Are you trying to lose weight now? (Baseline) | 0.7% | Mean imputation | 0.8 | 1.0 |
| Are you trying to lose weight now? (6 months) | 4.4% | Mean imputation | 0.9 | 1.0 |
| Self-efficacy score (Baseline) | 0.7% | Mean imputation | 29.8 | 30.0 |
| Social support and eating: Discouragement score (Baseline) | 0.7% | Mean imputation | 0.0 | 0.0 |
| How much can you rely on family and friends for support? (6 months) | 4.4% | Mode imputation | - | - |
| All weight efficacy subscores (6 months) | 7.3% | Multiple imputation | - | - |
| Self-efficacy score (6 months) | 8.8% | Multiple imputation | - | - |

*6.3 Code*

| **Method** | **Example Code** | **Description** |
| --- | --- | --- |
| **Creating data frame using ‘dplyr’** | library(dplyr)  variables <- intervention %>%  select(Num_medication, Race,  Gender, Age, Primary_diagnosis,  Weight_bln, Weight_6m,  Weight_efficacy_bln, …) | Using a dataset called “intervention”, we can use the select() function to choose the variables that we want to use in the subsequent analyses. |
| **Imputation using ‘mice’** | library(mice)  imputation <- mice(variables,  method=meth,  predictorMatrix=pred,  print = FALSE,m = 20,  seed= 22)  summary(imputation) | Using the mice() function, we can input a data frame of variables with missing entries. Each missing entry is filled with a plausible value based on a model using other variables in the data frame. We use method to select the imputation method (based on the measurement level of each column) and predictorMatrix to specify which other variables in the data frame can be used to predict the missing value of the variable of interest. m specifies the number of imputed datasets, and seed is to make sure we can reproduce the randomly generated results.^26^ |
| **Lasso using ‘MAMI’** | library(MAMI)  lasso <- mami(imputation,  model="binomial",  outcome="weight_binary_18mbl",  method="LASSO")  summary(lasso) | Using the mami() function, we can input the results of our imputation, which includes m=20 datasets. The function then performs Lasso logistic regression (method=”LASSO”), where we set the outcome and specify that it is binary using the model=”binomial” command. This regression is performed on each of the *m* datasets and coefficients are averaged across all models to determine a final estimate. |
| **Classification using ‘rpart’** | library(rpart)  imputation1<- mice::complete(imputation,1)  tree1 <- rpart(weight_binary_18mbl ~  Num_medication + Race +  Gender + Age + Primary_diagnosis +  Weight_bln + Weight_6m +  Weight_efficacy_bln + …,  data = imputation1,  method = "class")  plot(tree1, uniform=TRUE)  text(tree1, use.n=T, all=T, cex=.8) | First, we use the complete() function from ‘mice’ to fill in the missing values with the imputed results. Here, we use the first imputed dataset as an example. We can then use rpart() to make a classification tree. Here, we specify method=”class” so that we do a classification tree instead of a regression tree. The formula specifies that we want to fit a tree with the outcome of weight loss, and the branches can be based off of any of the variables that we list after the ~. Rpart() has rules with which it determines key splitting variables and values to determine branches, and the function ultimately outputs a classification tree that we can then plot and investigate. |

*6.46 Month Regression*

**Table A1.** Results from multiple logistic regression predicting weight loss (1) or gain (0) from baseline to 6 months.

|  | Estimate | Std. Error | t value | Pr(>\|t\|) |  |
| --- | --- | --- | --- | --- | --- |
| (Intercept) | 0.73 | 0.09 | 7.91 | 0.00 | *** |
| Black or African American | -0.19 | 0.10 | -1.96 | 0.05 | . |
| Asian | 0.01 | 0.31 | 0.05 | 0.96 |  |
| Native Hawaiian or other Pacific Islander | -0.08 | 0.37 | -0.22 | 0.83 |  |
| American Indian or Alaska Native | -0.34 | 0.24 | -1.45 | 0.15 |  |
| Female | -0.08 | 0.05 | -1.58 | 0.12 |  |
| Age | -0.05 | 0.04 | -1.17 | 0.25 |  |
| Baseline Weight | -0.01 | 0.05 | -0.12 | 0.90 |  |
| Schizoaffective Disorder | 0.03 | 0.11 | 0.23 | 0.82 |  |
| Bipolar Disorder | -0.11 | 0.14 | -0.82 | 0.41 |  |
| Major Depression | -0.01 | 0.15 | -0.06 | 0.95 |  |
| Other Diagnosis | -0.02 | 0.19 | -0.08 | 0.93 |  |
| Baseline Weight Efficacy Score | 0.03 | 0.05 | 0.65 | 0.52 |  |
| Any Antipsychotics | -0.09 | 0.05 | -1.86 | 0.07 | . |
| Baseline General Self Efficacy Score | -0.05 | 0.05 | -1.12 | 0.26 |  |
| Baseline MOS Social Support Score | 0.02 | 0.05 | 0.43 | 0.67 |  |
| Trying to Lose Weight at Baseline | 0.03 | 0.05 | 0.77 | 0.44 |  |
| Baseline Importance of Controlling Weight to Self | 0.01 | 0.05 | 0.32 | 0.75 |  |
| Baseline Confidence in Ability to Change to Control Weight | -0.04 | 0.05 | -0.71 | 0.48 |  |

*******p<0.01**.**p<0.10
